# Osteoblasts and Bone Marrow Mesenchymal Stromal Cells Control Hematopoietic Stem Cell Migration and Proliferation in 3D *In Vitro* Model

**DOI:** 10.1371/journal.pone.0009093

**Published:** 2010-02-08

**Authors:** Ana Paula D. N. de Barros, Christina M. Takiya, Luciana R. Garzoni, Mona Lisa Leal-Ferreira, Hélio S. Dutra, Luciana B. Chiarini, Maria Nazareth Meirelles, Radovan Borojevic, Maria Isabel D. Rossi

**Affiliations:** 1 Hospital Universitário Clementino Fraga Filho, Universidade Federal do Rio de Janeiro, Rio de Janeiro, Brazil; 2 Departamento de Ultrastructura e Biologia Celular, Instituto Oswaldo Cruz, Rio de Janeiro, Brazil; 3 Instituto de Biofísica, Universidade Federal do Rio de Janeiro, Rio de Janeiro, Brazil; Universidade do Porto, Portugal

## Abstract

**Background:**

Migration, proliferation, and differentiation of hematopoietic stem cells (HSCs) are dependent upon a complex three-dimensional (3D) bone marrow microenvironment. Although osteoblasts control the HSC pool, the subendosteal niche is complex and its cellular composition and the role of each cell population in HSC fate have not been established. In vivo models are complex and involve subtle species-specific differences, while bidimensional cultures do not reflect the 3D tissue organization. The aim of this study was to investigate in vitro the role of human bone marrow–derived mesenchymal stromal cells (BMSC) and active osteoblasts in control of migration, lodgment, and proliferation of HSCs.

**Methodology/Principal Findings:**

A complex mixed multicellular spheroid in vitro model was developed with human BMSC, undifferentiated or induced for one week into osteoblasts. A clear limit between the two stromal cells was established, and deposition of extracellular matrix proteins fibronectin, collagens I and IV, laminin, and osteopontin was similar to the observed in vivo. Noninduced BMSC cultured as spheroid expressed higher levels of mRNA for the chemokine CXCL12, and the growth factors Wnt5a and Kit ligand. Cord blood and bone marrow CD34^+^ cells moved in and out the spheroids, and some lodged at the interface of the two stromal cells. Myeloid colony-forming cells were maintained after seven days of coculture with mixed spheroids, and the frequency of cycling CD34^+^ cells was decreased.

**Conclusions/Significance:**

Undifferentiated and one-week osteo-induced BMSC self-assembled in a 3D spheroid and formed a microenvironment that is informative for hematopoietic progenitor cells, allowing their lodgment and controlling their proliferation.

## Introduction

Self-renewal and multilineage differentiation capacities that are dependent upon complex cell-autonomous and cell non-autonomous regulatory mechanisms are hallmarks of hematopoietic stem cells (HSC). In vivo studies have extensively documented the concept of a HSC niche, described as a three-dimensional microenvironment within the subendosteal region of bone marrow (BM) [1]–[3]. In this niche, HSC are protected from differentiation and loss of stem cell function possibly by induction of quiescence [4]. When they leave it, they enter into the transitional amplifying pool of committed progenitors, followed by terminal differentiation. However, HSC can exit the niche, circulate in blood, and eventually return to the BM niche. HSC homing to bone marrow is thus a physiological process [5], [6]. The role of several molecules such as the chemokine CXCL12 (SDF1-α), β1-integrins, and metalloproteinases in homing has been identified [7]-[9], but the complex interplays of cells and extracellular matrix (ECM) that allow some HSC to lodge at the subendosteal niche while others are actively mobile in the marrow cavity after intravenous injection [10], [11] are still puzzling. Furthermore, changes in the cellular composition of the niche modify the rate of HSC mobilization and homing [12]. Since the HSC niche was largely defined by their localization in marrow cavity, characterization of the stromal cell population within this niche and their role in the niche are still to be determined.

In the subendosteal niche, osteoblasts have been proposed to be a crucial component, controlling HSC fate, the size of HSC pool [13], [14], and HSC quiescence [15], by production of factors, such as angiopoietin-1 [16], CXCL12 [17], [18], and osteopontin [19], [20]. Cells of the sympathetic nerves [21] and osteoclasts [22] were recently described as important components of the niche. Furthermore, the subendosteal region is complex, harboring all cells that line at the interface between the bone surface and the marrow cavity, including stromal cells with differences in their osteogenic and myelopoietic supportive potential [23]. The endosteal surface of bones is covered not only by a heterogeneous cell population called bone lining cells [11], [24], but also by actively bone-producing osteoblasts [24].

Besides the subendosteal niche, HSC were also observed close to sinusoids, and the existence of a vascular niche was claimed [25], raising the question about the contribution of each niche to HSC regulation [26]. Trabecular bones are aligned with blood vessels [27] that are part of the bone remodeling compartment [28]. Recent data showed that the subendosteal region is also rich in blood vessels [11], [29], [30], suggesting that endothelial cells that were shown to contribute to hematopoiesis [31] might be part of the subendosteal niche. Blood vessel walls harbor a reserve of progenitor cells, known as mesenchymal stem cells or mesenchymal stromal cells [32]–[35]. Bone marrow-derived mesenchymal stromal cells (BMSC) exhibit the phenotype and anatomy of adventitial reticular cells [34] and organize marrow microenvironments when injected in vivo [34], [36], but their role in the subendosteal niche has not been fully studied. They were proposed to control HSC homing during ontogeny [37] and their renewal during adult life, phenomena that are dependent upon species-specific factors [38]. Despite the increasing knowledge on the HSC niche, central questions on the relative importance of each cell type, how they cooperate, as well as how HSC localize within the subendosteal niche which regulates their anchorage to osteoblasts require further analyses.

In vivo studies are restricted to animal models that might not reproduce species-specific subtleties. Although the contribution of conventional hematopoietic culture systems to the knowledge of human HSC biology is unquestionable, they are reductionist and do not reproduce the complex three-dimensional hematopoietic microenvironment. Striking differences in gene expression using bidimensional (2D) cultures as opposed to those obtained in vivo and in three-dimensional (3D) cultures have been reported [39]. The idea that mechanical and structural cues influence cell behavior and cell fate decisions is increasingly clear [40].

Our goal was to address the question of the relative participation of active osteoblasts and BMSC in the control of migration, anchorage, and proliferation of HSC. We have developed a 3D culture model with human BMSC that mimic the subendosteal region. We report that BMSC and osteo-induced BMSC assembled to form a well-organized multicellular spheroid where a clear boundary between these two cell populations were observed. Furthermore, human hematopoietic progenitor cells migrated dynamically in and out of these complex spheroids, where they lodged within discrete regions.

## Results

### Morphological Characterization of Simple and Mixed BMSC Spheroids

**Figure 1:**
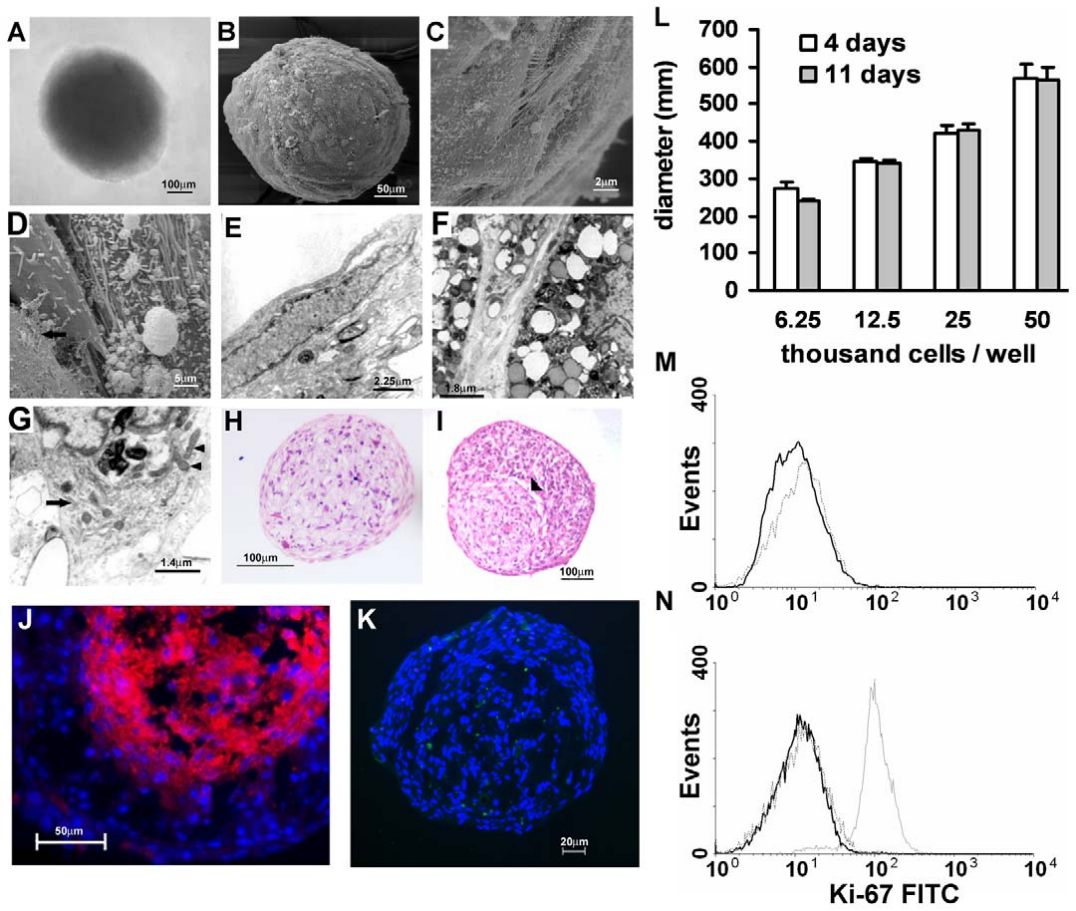
Characterization of simple and mixed spheroids. Simple non-induced spheroids are round cell aggregates (A–B) with elongated cells at the periphery (C, E) that show numerous cytoplasmic projections contacting neighbor cells (C), and delicate filaments (arrow) at the cell surface, indicative of ECM deposition (D). Inner cells have cytoplasmic vacuoles (F), irregular nucleus, well-developed rough endoplasmic reticulum (arrow), and many viable mitochondria (arrow heads) (G). Phase contrast (A), SEM (B–D), and TEM (E–G). Simple non-induced (H), and mixed (I) spheroids with osteo-induced BMSC at the center. Note the limit between the two cell populations (arrowhead). H–E. (J) Confocal microscopy of mixed spheroids confirming that CM-Dil labeled osteo-induced BMSC (in red) were confined to the central region. Nuclei were stained with DAPI (blue). (K) Detection of apoptotic cells (in green) in simple non-induced spheroids by TUNEL method after 5 days in culture. Nuclei were stained with DAPI (blue). (L) Correlation between the diameter of simple non-induced spheroids and the number of plated cells after 4 and 11 days of culture. Data represent mean of 3 spheroids per cell number per day. Error bars are standard error of the mean (± SEM). (M–N) Ki-67 expression in simple non-induced (M, full black line) and mixed (N, full black line) spheroids. Dotted lines are isotype control, and grey line (N) represents a cell line (porcine aortic endothelial cell) used as a positive control. Numbers above scale bars represent the value (in micrometers) of each scale bar.

**Figure 2:**
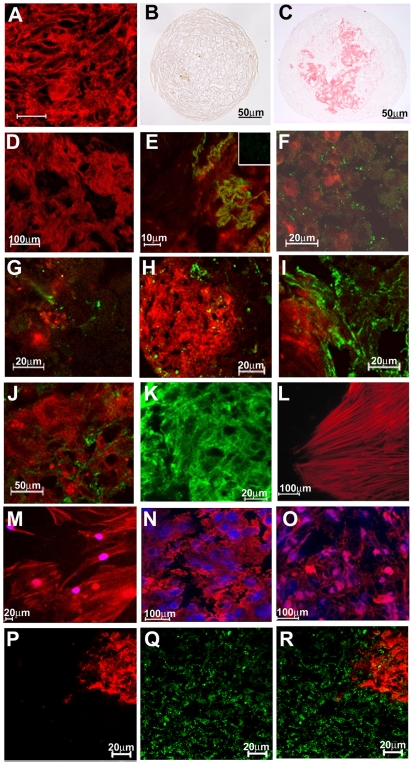
Extracellular matrix distribution and cytoskeleton organization in simple non-induced and mixed spheroids. (A) Confocal microscopy of paraffin sections stained with H&E showing complex cellular interactions in the center of simple non-induced spheroids. (B–D) Picrosirius staining of simple non-induced (B) and mixed (C–D) spheroids showing, by optical (B–C) and confocal (D) microscopy, collagen fiber deposition restricted to the inner region of mixed spheroids. (E–J) Expression of ECM protein in mixed spheroids. Immunofluorescence staining (in green) for collagen I (E), laminin (F), collagen IV (G), osteopontin (H), and fibronectin (I–J). Osteo-induced BMSC were labeled with CM-DiI (red). A negative control is shown as an insert in (E). (K) Fibronectin expression in simple non-induced spheroids is shown for comparison. (L–O) Expression of α-SMA (L, N) and actin polymerization (M, O, phalloidin staining) in non-induced BMSC. Note the formation of stress fibers in monolayers (L–M) that are absent in 3D cultures (N–O). Nuclei were stained with DAPI (blue). (P–R) α-SMA expression (green) in mixed spheroids. Osteo-induced BMSC were labeled with CM-DiI (red in P, R). Numbers above scale bars represent the value (in micrometers) of each scale bar.

Considering that the three-dimensional structure of bone marrow may be important for the organization of specialized niches, undifferentiated (non-induced) BMSC were cultured on a non-adherent surface that favors cell aggregation. After 4 days, BMSC formed spheroid structures (simple non-induced spheroids) of approximately 400 µm diameters (420 µm ± 23.7) (Fig. 1A–B). Scanning electronic microscopy showed that cells with numerous filopodia-like projections contacted neighboring cells (Fig. 1C) and delicate filaments indicative of extracellular matrix (ECM) (Fig. 1D, arrow) formed a smooth external layer (Fig. 1B). Histological and ultrastructural sections of simple non-induced spheroids confirmed the fusiform aspect of cells at the periphery (Fig. 1E, H). Inner cells were polyedric (Fig. 1H) and had irregular nuclei, vacuolized cytoplasms, well-developed rough endoplasmic reticulum and many mitochondria (Fig. 1F–G). Cytoplasmic projections contacting neighboring cells (Fig. 1F) formed a complex three-dimensional network (Fig. 2A) similar to reticular cells in vivo [41]. The intercellular space was filled with a granular material suggestive of ECM deposition (Fig.1F). Osteo-induced BMSC formed similar spheroids, called simple osteo-induced spheroids (data not shown). Thus, human BMSC, were able to reassemble in a complex 3D network similar to reticular cells in the marrow cavity [41].

We were then able to develop mixed spheroids formed by osteo-induced and non-induced BMSC. The aim was to create specialized regions, and by surrounding osteo-induced spheroids with BMSC we sought to mimic the trabecular bone that is also surrounded by reticular cells. Furthermore, homing of HSC involves migration throughout a three-dimensional meshwork of cytoplasmic projections of stromal cells, followed by lodgment at the subendosteal niche [1], [8]. Since cells of the osteoblast lineage within the endosteal niche are heterogeneous [23], [24] and differ in their capacity to support hematopoiesis [23], [36], BMSC were osteo-induced in vitro for just one week. Osteo-induced cells were thus plated to form an inner spheroid that was covered with layers of non-induced BMSC. In fact, non-induced BMSC were found around the spheroids formed by osteo-induced BMSC. Clear boundaries were observed between the two cell populations (Fig. 1I–J).

In tumor spheroids, cell proliferation is seen at the periphery, and a central necrotic area is observed in spheroids larger than 400–500 µm [42] due to critical O_2_ concentration in the central region [42], [43]. Cell death may lead to a decrease in the diameter of spheroids along time [44]. However, the size of simple non-induced (Fig. 1L) and of mixed spheroids correlated with numbers of plated cells and did not change with time in culture. We then investigated the frequency of cell proliferation and death in the spheroids. In simple non-induced spheroids, cell death reached 11.1% (± 6.43) (Fig. 1K) after 5 days in culture while in mixed spheroids 18.1% (± 7.1) were observed. Cell proliferation in simple non-induced and mixed spheroids was evaluated by Ki-67 staining. In agreement with in vivo descriptions [45], almost all the BMSC cells were quiescent (Fig.1M–N). Distribution of ECM proteins in bone marrow is well known, so we investigated the pattern of ECM in mixed spheroids. Similarly to what has been described in vivo [46], collagen I fibers, the major ECM protein of bone, were observed exclusively in the central region formed by osteo-induced BMSC (Fig. 2C–E). No collagen fibers were present in simple non-induced spheroids (Fig. 2B). Low amounts of laminin (Fig. 2F), and collagen IV (Fig. 2G), but high levels of fibronectin (Fig. 2I–J) were present in central and peripheral regions, as well as in simple non-induced spheroids (Fig. 2K). Since osteopontin is observed exclusively in the interface between bone and marrow cavity [20], and it has a fundamental role in the subendosteal niche [19], [20], its expression in mixed spheroids was also investigated. Osteopontin was expressed at low levels, mostly at the interface between osteo-induced and non-induced BMSC (Fig. 2H), suggesting that mixed spheroids reproduced the subendosteal ECM protein pattern.

Human BMSC constitutively express α-smooth muscle actin (α-SMA) both in vitro [47] and in vivo [45]. However, it was shown that α-SMA^+^ fibroblasts in 2D cultures lose this marker when cultured in 3D systems such as multicellular spheroids [48]. Comparison between our 2D and 3D cultures, showed that non-induced BMSC are α-SMA^+^ in both culture systems (Fig. 2L, N) although differences in orientation of the filaments were observed. While stress fibers were present in cells cultured in 2D (Fig. 2L–M), they were absent in cells that formed 3D spheroids as in vivo (Fig. 2N–O). Pre-osteoblastic differentiation into more mature stages was shown to be associated with a decrease in α-SMA [49]. We observed that in mixed spheroids most osteo-induced BMSC were negative for α-SMA (Fig. 2P–R), suggesting that one-week of induction in vitro lead to differentiation of BMSC into α-SMA^−^ collagen I-secreting osteoblast. We concluded that fresh and osteo-induced BMSC can be induced to form 3D structures in culture, with many features previously described for normal marrow stromal cells in situ.

### Differential Gene Expression in BMSC Cultured in 2D or 3D

**Figure 3:**
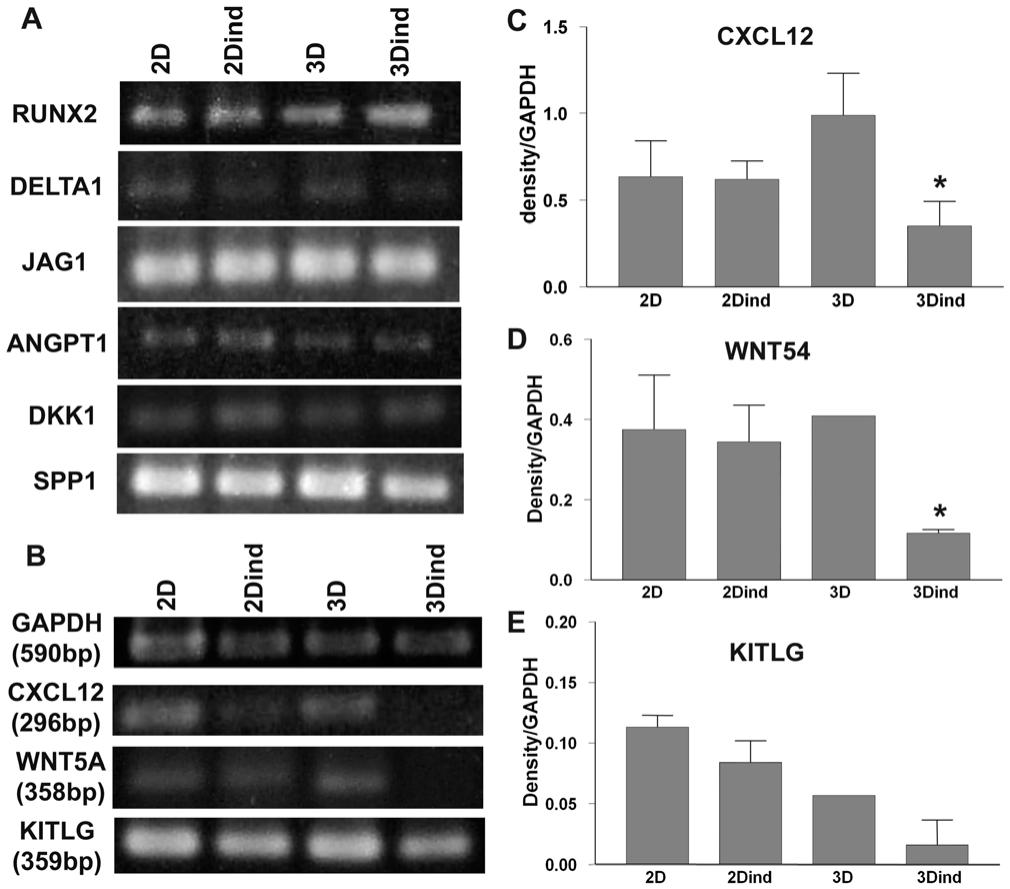
Gene expression profile of BMSC in 2D or 3D cultures. (A) A representative RT-PCR analysis is shown for osteo-induced BMSC cultured for 4 days as monolayers (2D ind) or as simple osteo-induced spheroids (3D ind), and non-induced BMSC cultured as monolayers (2D) or simple non-induced spheroids (3D). Blots correspond to the transcriptional factor RUNX2, the Notch ligands DELTA1 and JAG1 (Jagged-1), ANGPT1 (Angiopoietin-1), the inhibitor of Wnt pathway, DKK1, and SPP (Osteopontin). (B–E) Semiquantitative analysis is shown for GAPDH, CXCL12 (C), WNT5a (D), and KITLG (E). (n = 3, ± SEM).

Gene expression pattern differ depending on whether the cells are grown in a 2D or in a 3D substrates [39]. We sought to investigate the expression of the transcription factor RUNX2 (CBFA1), which is involved in osteoblast differentiation [24], and of genes that control HSC properties in the niche [2], [3]. Non-induced as well as osteo-induced BMSC expressed RUNX2, Notch ligands, DELTA1 and JAG1 (Jagged1), ANGPT1 (Angiopoietin1), SPP1 (Osteopontin), and the Wnt inhibitor DKK1 (Fig. 3A). No differences were observed regarding the culture condition. However, non-induced BMSC expressed higher levels of CXCL12 (Fig. 3B–C), WNT5a (Fig. 3B, D), and KITLG (Kit-ligand, SCF) (Fig. 3B, E), and the difference was even more evident in simple non-induced spheroids compared to simple osteo-induced ones. These findings suggested that in BMSC normal patterns of gene expression are unlikely to be achieved in conventional adherent cell cultures and that geometry influences stromal cell differentiation.

### Hematopoietic Progenitor Cells Migrate into Simple and Mixed Spheroids

**Figure 4:**
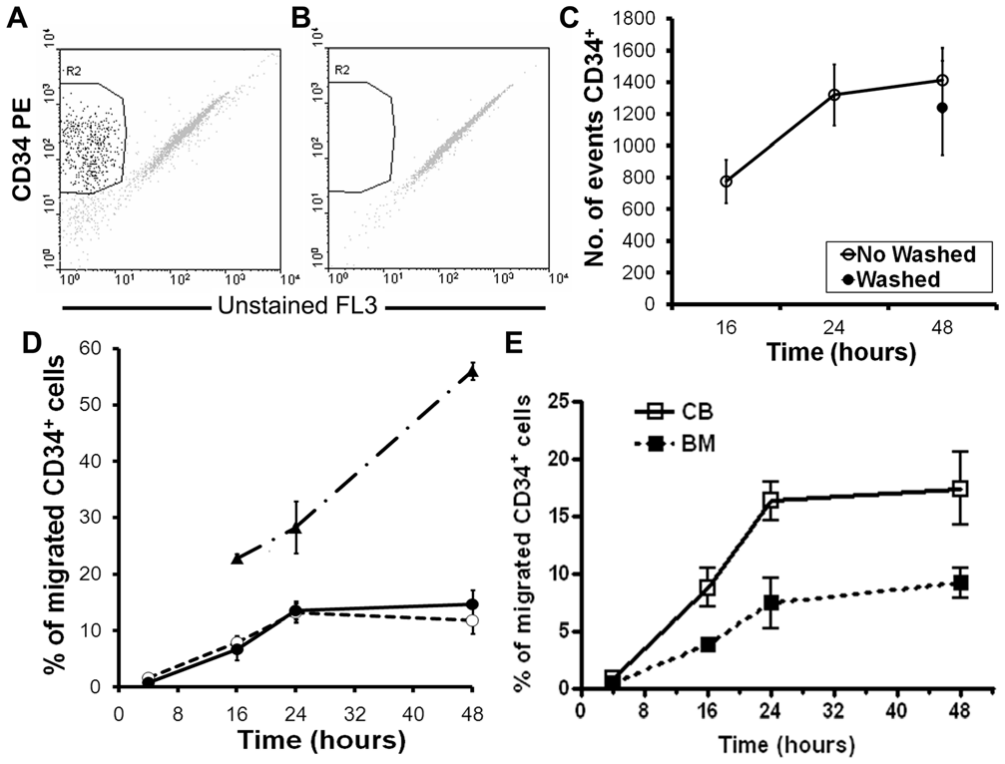
Time-dependent migration of CD34^+^ cells into spheroids. At defined intervals, cells in the supernatant were collected and spheroids were harvested and trypsinized. (A) The proportion of CD34^+^ cells (R2) was determined by flow cytometry, and calculated as percentages of CD34^+^ cells inside the spheroids in relation to the total plated. (B) Dot plot of control spheroids without hematopoietic cells. (C) To distinguish migration from proliferation of cells inside the spheroids, hematopoietic cells were removed after 24 hours of co-culture, and the spheroids were maintained in culture for up to 48 hours. The number of CD34^+^ events inside the washed spheroids (closed symbols) was compared to the number of CD34^+^ events inside no washed spheroids (open symbols). (D) Time-dependent migration of CB CD34^+^ cells into simple non-induced (full line, dots), simple osteo-induced (dotted line, triangles), and mixed (dotted line, circles) spheroids. (E) The migratory profile of BM (closed squares) and CB (open squares) CD34^+^ cells in mixed spheroids is shown. Data are mean ± SEM.

Homing of transplanted HSC to the host marrow is a multi-step process that involves trans-endothelial migration, followed by adhesion to the marrow stroma and migration towards the specific anatomical sites [7], [8]. Therefore, we asked whether hematopoietic cells could invade and physically interact with our in vitro model. In order to investigate the role of each BM stromal cell population and their interaction in the control of migration and location of hematopoietic stem and progenitor cells, magnetically selected cord blood (CB) or bone marrow (BM) CD34^+^ cells were co-cultured with simple non-induced, simple osteo-induced, and with mixed spheroids. CD34^+^ cells were seen in enzymatically digested spheroids by FACS analysis (Fig. 4A). No CD34^+^ events were found in control spheroids that were not co-cultured with hematopoietic cells (Fig. 4B). To distinguish migration from proliferation inside the spheroids, hematopoietic cells were removed after 24 hours of co-culture, the spheroids were vigorously washed, and maintained for up to 48 hours. The content of CD34^+^ events in washed (1239 ± 297.8) and no-washed (1412.3 ± 202.7) spheroids was quite similar (p = 0.309) after 48 hours of culture (Fig. 4C). This suggests that no significant proliferation of CD34^+^ cells occurs within 48 hours of co-culture with mixed spheroids. Migration of CD34^+^ cells was seen as early as 4 hours after initiation of co-cultures with simple non-induced and mixed spheroids and increased thereafter, reaching a plateau around 24 hours (Fig. 4D). Conversely, in simple osteo-induced spheroids the cells migrated continuously (Fig. 4D).

Various sources of CD34^+^ cells are known to differ in their capacity to engraft immunocompromised mice. For example, CB CD34^+^ cells engraft in vivo more rapidly and efficiently than BM CD34^+^ cells [50]. We investigated whether our 3D in vitro model would reproduce these differences, and indeed, BM CD34^+^ cells migrated less efficiently than CB CB34^+^ cells at 16 (p = 0,046) and 24 hours (p = 0,011) (Fig. 4E). Thus, migration into BMSC spheroids was not random, but depended upon the properties of CD34^+^ and stromal cells.

### Migration of Hematopoietic Progenitor Cells into Spheroids Is Dynamic

**Figure 5:**
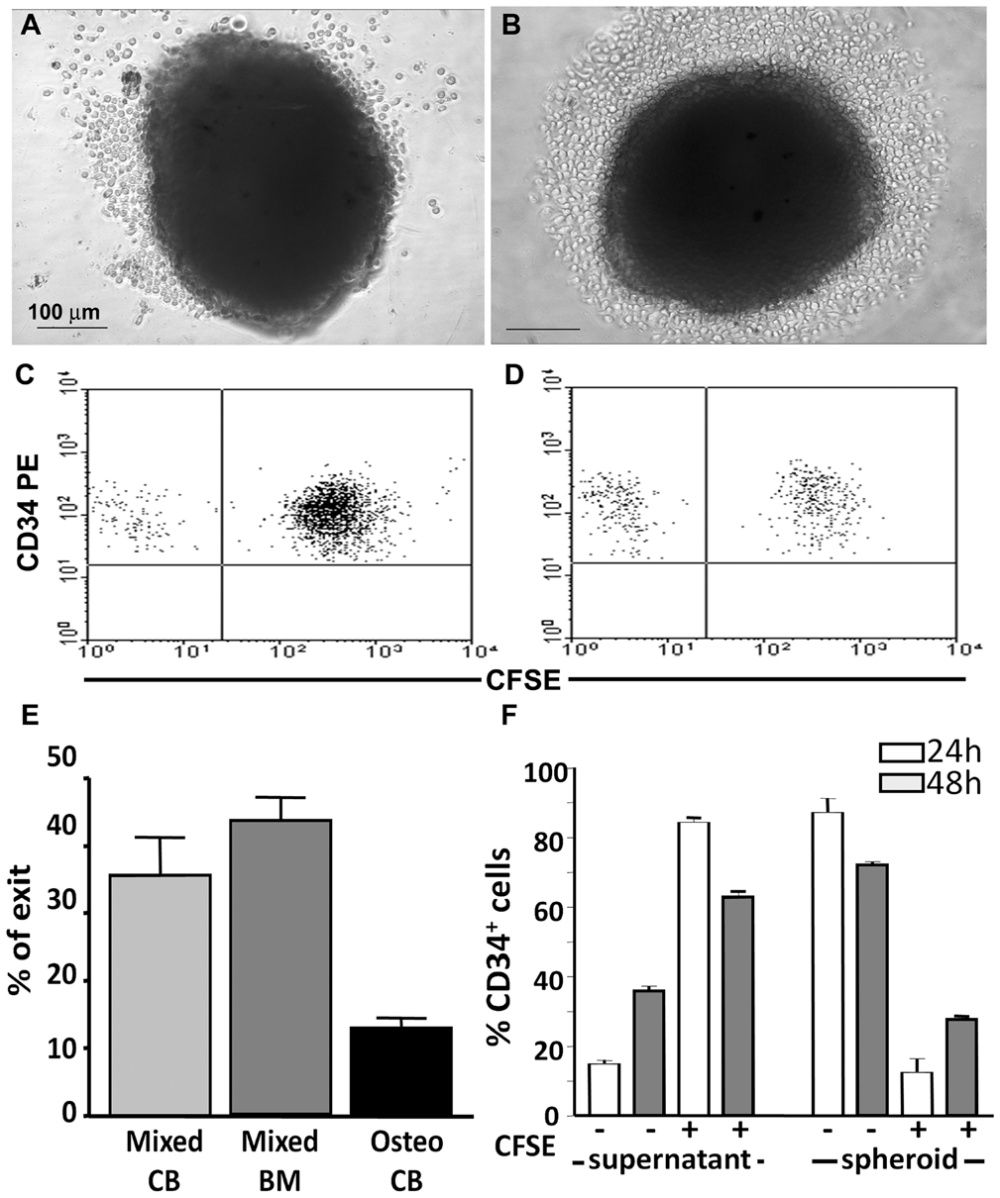
Migration of CD34^+^ cells in mixed spheroids is dynamic. CB and BM CD34^+^ cells were co-cultured with simple or mixed spheroids for 24 hours. The supernatant was removed and the spheroids were washed and maintained in culture for more 48 hours. Phase contrast microscopy of CB CD34^+^ cells emigrating from mixed spheroids at 24 hours (A) and 48 hours (B). Number above scale bar represents the value (in micrometers) of both scale bars. Percentage of CB and BM CD34^+^ cells that migrated out from mixed and simple osteo-induced spheroids was determined by flow cytometry (E). Unlabeled cells were co-cultured with mixed spheroids for 24 hours and then the supernatants were removed and the spheroids were washed. CFSE labeled CD34^+^ cells were added to these spheroids and the co-cultures were maintained for an additional 48 hours. Representative FACS analysis showing CD34^+^ cells that were CFSE^−^ or CFSE^+^ in supernatants (C) and spheroids (D) after 48 hours of co-culture. (F) Histogram showing the percentage of CD34^+^ cells that were positive or negative for CFSE in the supernatant or spheroids after 24 (open bar) and 48 hours (grey bar). Data represent average percentages (± SEM) of CFSE^+^ and CFSE^−^ among CD34^+^ cells in one experiment with duplicates. Similar results were obtained in a second independent experiment.

After BM transplantation, HSC migrate in and out the marrow within hours [10]. As shown above, in our 3D model, migration of CD34^+^ reached a plateau after 24 hours of culture with mixed and simple non-induced spheroids, but not with simple osteo-induced spheroids (Fig. 4D). We questioned whether differences in adhesion to non-induced versus osteo-induce BMSC were leading to the migration of CD34^+^ cells in and out of the spheroids. CD34^+^ cells were co-cultured for 24 hours with spheroids that were subsequently washed and maintained in culture. After 24 (Fig. 5A) and 48 hours (Fig. 5B), CD34^+^ cells migrated out the mixed spheroids, with no differences between CB and BM CD34^+^ cells (Fig. 5E). However, CB CD34^+^ cells migrated out from simple osteo-induced spheroids to a lesser extent (Fig. 5E), explaining the absence of a plateau during 48 hours of culture. Virtually no BM CD34^+^ cells migrated out of simple osteo-induced spheroids (data not shown).

We then investigated whether CD34^+^ cells were actively moving in and out of mixed spheroids. Unlabeled CB CD34^+^ cells were cultured with mixed spheroids for 24 hours and washed out. Since no non-adherent cells were observed in the supernatant, CFSE labeled CB CD34^+^ cells were added, and the cultures were maintained for up to 48 hours. The percentage of CD34^+^ cells that were positive or negative for CFSE in the supernatant and spheroids was determined by FACS (Fig. 5C–D). Although no changes in the total number of CD34^+^ events were observed (data not shown), an inverted relationship between labeled and unlabeled cells in spheroids and supernatants was seen along the culture period (Fig. 5F). At 24 hours, the proportion of unlabeled cells in relation to CFSE^+^ cells in the supernatant was 1∶8 and the inverse (8∶1) was observed inside the spheroids. At 48 hours, the proportion changed to 1∶2 (unlabeled CD34^+^ cells: CFSE^+^ cells) in the supernatant, whilst the exact opposite in the spheroids (2∶1) was seen. Such a tight correlation is not explained by cell death or proliferation. Moreover, CFSE labeling showed that the mean fluorescence intensity of the dye did not change within 48 hours (data not shown) and no increase in CD34^+^ events inside spheroids that were washed was observed after 48 hours of co-culture (Fig. 4C). Taken together, the data suggest that CD34^+^ cells moved in and out the mixed spheroids in a very dynamic way. Furthermore, since a higher retention was observed in simple osteo-induced spheroids that lack an outer layer of undifferentiated BMSC, these data suggest that the quality of hematopoietic progenitor cells adhesion to the stromal cells or ECM components control their migration.

### Mixed Spheroids Mimic Subendosteal Niches in BM

**Figure 6:**
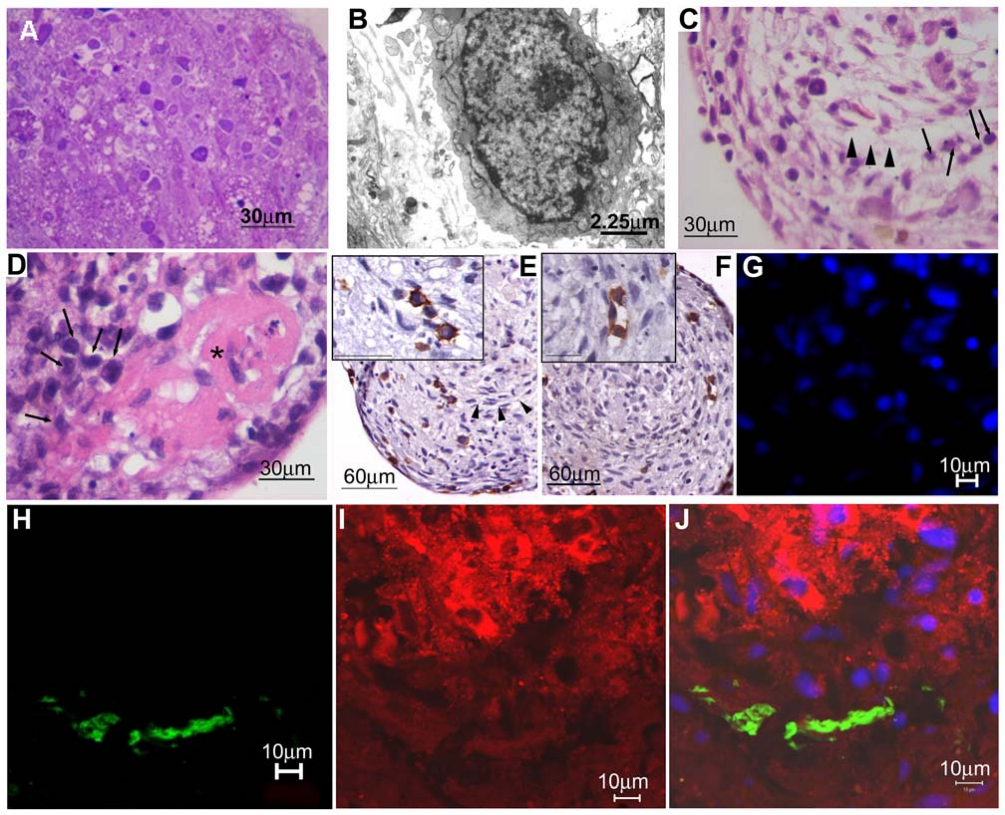
CD34^+^ cells localize at the vicinity of osteo-induced BMSC in mixed spheroids. (A) Semithin section of simple non-induced spheroids after 48 hours of co-culture with CB CD34^+^ cells, showing numerous hematopoietic cells homogeneously distributed throughout the spheroids, even at the center. Methylene Blue. (B) Ultrastructure of the co-cultures, showing a CD34^+^ cell in contact with stromal cell projections. (C–D) Mixed spheroids co-cultured for 72 hours with CB CD34^+^ cells. Hematopoietic cells (arrows) were aligned at the interface of the two stromal cell layers (C, arrowhead) or at the vicinity of osteoid tissue (* in D). H–E. (E–F) Clusters of CD34^+^ cells (inserts) are seen at the vicinity of the osteo-induced BMSC after 48 hours. Immunohistochemistry. (H–J) Confocal microscopy of mixed spheroids co-cultured for 72 hours with CFSE labeled CB CD34^+^ cells (green, H, J). Osteo-induced BMSC were labeled with CM-DiI (red, I–J) and nuclei, that was not confocalized, were stained with DAPI, (blue, G, J). Note that hematopoietic cells are located in close proximity to osteo-induced CM-Dil^+^ BMSC but actually at the transitional region between the two cell populations. Numbers above scale bars represent the value (in micrometers) of each scale bar. (Bars in inserts  = 30 µm).

In BM, HSC home to and lodge within the subendosteal niches [1]. Since a very clear zone limiting the central region of osteoblasts and the peripheral area of non-induced BMSC was established in mixed spheroids, we investigated whether CB CD34^+^ cells would recognize and localize specifically in this area. CB CD34^+^ cells cultured for 48 hours with simple non-induced spheroids were homogenously distributed among the stromal cells, even in the central regions (Fig. 6A–B). The same was observed in cultures with simple osteo-induced spheroids (data not shown). However, in mixed spheroids, hematopoietic cells were initially observed at the periphery, but within 48 hours they were close to the central region (Fig. 6C–F). Non-mineralized osteoid tissue was occasionally formed in this type of cultures and hematopoietic cells surrounded it (Fig. 6D). It is striking that although CB CD34^+^ cells were able to migrate throughout the simple osteo-induced spheroids, no CD34^+^ were found in the central region of mixed spheroids. This localization of CB CD34^+^ cells was confirmed by immunohistochemistry and confocal microscopy (Fig. 6E–J). CB CD34^+^ cells were found in clusters at the vicinity of the central area formed by osteo-induced BMSC, as it has been described in vivo [1], [11], [30], suggesting that mixed spheroids formed a microenvironment that is representative of the subendosteal niche, identified as such by human hematopoietic progenitor and stem cells.

**Figure 7:**
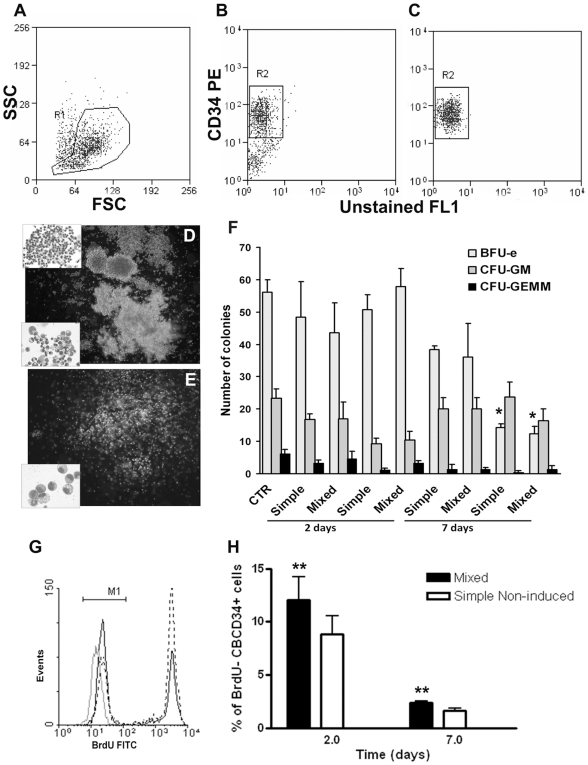
Colony formation in Methylcellulose and BrdU incorporation. The sorted CD34^+^ cells from the supernatant or from inside simple non-induced and mixed spheroids after 2 and 7 days of culture were subjected to methylcellulose assay to determine their potential clonal growth as CFU-GEMM, BFU-e and CFU-GM. (A–C) Representative profile of single-cell suspension isolated from co-cultures before (A–B) and after sorting of CB CD34^+^ cells (C). (D–E) Morphology of day-10 colonies (inverted microscope, x 5); inserts shows cellular morphology of individual colonies (MGG staining, x 40 in D, x 60 in E). (F) Clonogenic myeloid potential in methylcellulose of CD34^+^ freshly isolated from CB or co-cultured for 2 and 7 days with mixed or simple non-induced spheroids (* p = 0.0015). Data is representative of two independent experiments with triplicates. Mean ± SEM. (G) FACS analysis of BrdU expression in CB CD34^+^ cells co-cultured for 48 hours with simple non-induced (dotted line) or mixed spheroids (black line). The grey line represents isotype control. (H) The percentage of CD34^+^ cells that were negative for BrdU after co-culture for 2 or 7 days with simple non-induced (grey bars) and mixed (white bars) spheroids is shown. (** p = 0.037; n = 4 ± SEM).

Proliferation and differentiation potentials of cultured CB CD34^+^ cells were evaluated by colony assays. To avoid contamination with stromal cells, CD34^+^ cells were sorted after 2 and 7 days of co-culture with simple non-induced or mixed spheroids (Fig. 7A–C) and plated into methylcellulose. We found that the frequency of CFU-c was quite similar to the freshly isolated CB CD34^+^ cells, although a significant (p = 0.0015) reduction in colony forming cells was observed after 7 days. This was mainly due to the loss of BFU-e inside simple non-induced and mixed spheroids but not in the supernatants (Fig. 7D–F). Some CFU-GEMM were recovered from spheroids even after a week of culture.

HSC residing in bone marrow divide regularly but infrequently [51], so we investigated this parameter in our culture model. CB CD34^+^ cells were cultured with mixed or simple spheroids in the presence of BrdU and incorporation of this label was determined by FACS (Fig. 7G). The percentages of CD34^+^ cells that did not incorporate BrdU after 2 and 7 days of culture were significantly higher (p = 0.037) in mixed spheroids (Fig. 7H), implying that BMSC osteo-induced for just one week are able to restrain hematopoietic progenitor cell proliferation in this 3D microenvironment. Taken together, the data suggest that association of BMSC and active osteoblasts in a 3D structure form an informative microenvironment that control migration, lodgment, and proliferation of hematopoietic progenitor and stem cells.

## Discussion

HSC niche have been defined based on HSC localization within the subendosteal region with poor description of the neighboring cells and their role in the control of HSC niche function. Osteoblasts and more recently blood vessel endothelial cells have been proposed to be key elements in the HSC niche. Here, we show that non-induced and one-week osteo-induced human bone marrow stromal cells self-reassembled in vitro in 3D spheroids, and they are required and sufficient to control migration, lodgment and proliferation of human hematopoietic progenitor cells. These two cell populations developed a microenvironment in vitro that reproduced some properties of the subendosteal niche. Bone marrow stromal cells became quiescent, organized themselves forming a meshwork, deposited extracellular matrix, and formed specialized microenvironments that were recognized by hematopoietic CD34^+^ cells.

Perivascular BMSC are α-SMA^+^ quiescent cells [45] with cytoplasmic projections that form a 3D meshwork and closely envelop hematopoietic cells [41]. Conventional culture systems disrupt these features and induce cytoskeleton reorganization and stress fibers [48] that could modulate cell differentiation [52]. Here we show that in 3D spheroid cultures, without any scaffold, BMSC rearranged their cytoskeleton, lost stress fibers, became quiescent, and emitted numerous membrane projections. Furthermore, the distribution of ECM proteins, fibronectin, laminin, collagen I and IV reproduced the in vivo situation [46], with collagen I at the central region formed by osteo-induced BMSC, very little laminin and collagen IV, and a wide distribution of fibronectin. Osteopontin was observed mostly at the interface between the two cell populations. Since its expression was shown to be restricted to the subendosteal niche [19], [20], we propose that mixed spheroids mimic the ECM distribution of the subendosteal niche.

Long-term engraftment of HSC depends upon homing, transmarrow migration and lodgment of HSC within a specific niche. Mixed and simple non-induced spheroids initially elicited CD34^+^ cell migration with similar kinetics. However, the outwards migration was rapidly established, reaching already after 24 hours the steady state equilibrium of cells retained in the spheroid with the free cell pool, indicating equal input and output. On the other hand, human CB and BM CD34^+^ cells migrated continuously into the osteo-induced BMSC spheroids and were not released, indicating that adhesion was stronger than cell motility, retaining the cells within the spheroid. These phenomena are apparently specific of the cell origin, since CB CD34^+^ cells homed more efficiently than BM CD34^+^ cells in immunocompromised mice [50], and species-specific, since human hematopoietic progenitor cells were reported to migrate continuously into murine bone marrow stroma spheroids [53]. The entrance and exit of progenitors in mixed spheroids is similar to the natural maintenance of blood cell homeostasis and during regeneration of hematopoietic bone marrow after intravenous injection of blood cell progenitors [10].

HSC lodgment within the subendosteal niche [1]–[3], [18] is dependent upon intrinsic features and microenvironment cues, such as angipoietin-1 and CXCL12, provided by osteoblasts [11], [13]–[18], [22]. However, osteoblast lineage cells that are found within the subendosteal niche include osteoprogenitor cells, quiescent bone lining cells, and active osteoblasts [11], [24]. Besides, recent data showed that the subendosteal niche is perivascular and a role for endothelial cells in HSC fate decisions was proposed [11], [21], [22]. However, mesenchymal stromal cells are located in the perivascular niche [32]–[35] and they secrete an array of cytokines and chemokines, including angiopoietin-1 [34]. In mice with the GFP reporter gene knocked into the CXCL12 locus, expression of this chemokine was observed in perivascular reticular cells that were also α-SMA^+^ [54]. We observed that BMSC were α-SMA^+^ in spheroids and had more CXCL12 transcripts as compared to osteo-induced cells, especially in a 3D microenvironment. This is in agreement with the idea that gene expression is modulated in response to geometric and mechanical cues [39]. Moreover, since CXCL12 gene expression is regulated by hypoxia-inducible fator-1 (HIF) [55], [56], the hypoxic milieu of spheroid cultures [42], [43] might mimic the low perfusion regions of BM [29], [57] and increase CXCL12 production by BMSC, which is currently under investigation. This difference in CXCL12 expression raises the hypothesis that BMSC have essential role in establishing a chemokine gradient that controls HSC engraftment and location inside the marrow cavity. Since accumulation of human CD34^+^ cells in a specific region was only observed in mixed spheroids, we propose that both BMSC and active osteoblasts are needed to establish an informative microenvironment that allows positioning of human CB CD34^+^ cells. This specific location of HSC was not observed in simple non-induced or simple osteo-induced spheroids where human CD34^+^ cells were randomly distributed among stromal cells. Cross-talk between the two phenotypes of cells composing the niche is apparently required, and in our model it was sufficient to control the positioning and proliferation of hematopoietic progenitors. The lodgment of HSC within the subendosteal region is probably determined by opposing gradients of factors associated with osteoblasts, such as calcium ions [3], [58], and those produced by BMSC, such as CXCL12. Although calcium deposits were only rarely observed in these mixed spheroids (data not shown), it is possible that higher concentrations of this ion exist in the central region. HSC adhere to collagen I [58], which is secreted by osteo-induced BMSC in mixed spheroids. Adhesion of CD34^+^ cells to collagen I could contribute to the localization of these cells at the vicinity of osteo-induced cells and to their retention inside simple osteo-induced spheroids. Furthermore, OPN, that attracts and retains HSC to the endosteal surface [20], was also predominantly deposited at the interface between the two stromal cell populations in mixed spheroids. Although the BMSC were osteo-induced for just one week, they were able to display some properties of the subendosteal environment in agreement with other findings in the literature [36], [59]. The fact that deletion of osteoblasts under the control of collagen α1 type I promoter, but not osteocalcin promoter, is followed by a decrease in the numbers of HSC [59] suggests that osteoblasts that have not reached terminal differentiation are important organizers of the subendosteal niche. Our findings support this hypothesis. In mixed spheroids, one-week osteo-induced BMSC lost the expression of α-SMA^−^, were actively secreting collagen-I, and were sufficient to reduced proliferation of hematopoietic cells that interact with them.

Another aspect that deserves comments is the role of hypoxia in regulating HSC function [29], [55]. Although the low oxygen tension in the inner regions of spheroids [42], [43] might mimic the low perfusion regions of BM [29], [57] and regulate CXCL12 gene expression [55], [56] as already pointed out, it also might result in the observed decrease in BFU-e content after 7 days. In the absence of erythropoietin, hypoxia suppresses erythroid progenitor/precursor cell proliferation and differentiation [60], [61]. The absence of those progenitors compromised the read-out of the colony assay since numbers of early progenitors are based on the presence of CFU-GEMM. In order to clarify this point SCID repopulating or Long-Term Culture - Initiating Cell (LTC-IC) assays should be done.

In conclusion, non-induced and one-week osteo-induced BMSC self-assembled as a 3D spheroid formed an informative microenvironment that allows migration and lodgment of hematopoietic progenitor and stem cells. Osteo-induced BMSC restrain migration and proliferation of hematopoietic CD34^+^ cells. It will be important to learn if requirements for self-renewal are satisfied, and if that is the case we can construct even more complex environments by including endothelial and other cell types to investigate their role in HSC fate.

## Materials and Methods

### Cells and Sample

Human skin fibroblasts (HSF) and porcine aortic endothelial cell (PAEC) were obtained from the Rio de Janeiro Cell Bank (BCRJ, Rio de Janeiro, Brazil). Human cord blood (CB) samples were obtained from healthy full-term newborns at the Pro-Matre Hospital (Rio de Janeiro, RJ, Brazil). Bone marrow samples were obtained from voluntary donors at the Bone Marrow Transplant Unit, Hematology Service, Hospital Universitário Clementino Fraga Filho (HUCFF) at UFRJ by washing with phosphate buffered saline (PBS) the bone marrow collection kits after bone marrow aspirates were transferred to infusion bags. Informed consent exemption was approved by the Investigational Review Board at HUCFF since data were analyzed anonymously and derived from discarded samples.

### Antibodies

Fluorescein (FITC) or R-phycoerythrin (PE) conjugated monoclonal mouse anti-human antibodies, CD45 (HI-30), 5-bromo-2′-deoxyuridine (BrdU; B44), Ki-67 (mib-1, B56), anti-CD34 (HPCA-2, 8G12) were purchased from BD-Pharmingen (San Jose, CA). Unlabeled mouse anti-α-smooth muscle actin (Dako, Glostrup, Denmark), anti-CD34 (QBEND10, Biogenex, San Ramon, CA), and anti-collagen I and IV, rabbit anti-fibronectin and anti-laminin (all from Sigma-Aldrich, St. Louis, MO), and goat anti-osteopontin (Santa Cruz Biotech. Inc., Santa Cruz, CA) were used for immunofluorescence or immunohistochemistry. Primary antibodies were revealed by secondary reagents, tetramethylrhodamine (TRITC) conjugated goat anti-mouse IgG (Sigma-Aldrich), Alexa 488 conjugated rabbit anti-mouse IgG and donkey anti-goat IgG (Invitrogen-Molecular Probes, Carlsbad, CA), and FITC anti-rabbit IgG, F(ab)_2_ fragment (Boehringer Mannheim, Mannheim, Germany).

### Isolation of CD34^+^ Cells and Stromal Cells

Mononuclear cells were collected from CB or adult BM by Ficoll-Hypaque Plus™ (GE Healthcare Life Sciences, São Paulo, SP, Brazil) centrifugation. Enrichment for CD34^+^ cells was performed using immunomagnetic beads (CD34 Progenitor Cell Selection System, Dynal Biotech, Lake Success, NY), following manufacturer's instructions. The purity of CD34^+^ cells obtained from CB and BM was respectively 60–90% (median  = 78.7) and around 90%, as described by the manufacturers. Bone marrow-derived stromal cells (BMSC) were obtained and maintained as previously described [62]. Briefly, hemo-sedimentation was performed by diluting (6∶1) BM aspirates with Hespan® (hydroxyethyl starchhaline, American Hospital Supply Corp., McGaw Park, IL). After 30 minutes at room temperature (RT), the supernatant was collected, washed with PBS and 1×10^6^ cells/mL were plated in 25 cm^2^ flasks with Dulbecco's low-glucose medium (DMEM Low-glucose, LGC, São Paulo, SP, Brazil) supplemented with 10% fetal bovine serum (FBS, Cultilab, Campinas, SP, Brazil) and antibiotics (100 U/ml of penicillin and 100 µg/ml of streptomycin, both from Sigma-Aldrich). After 3 days, non-adherent cells were removed, and the adherent cells were washed twice with PBS. Fresh complete medium was added and cells were cultured for up to 21 days. Adherent cells were harvested with 0.125% trypsin and 0.78 mM EDTA (Sigma-Aldrich).

### In Vitro Differentiation of BMSC Toward the Osteoblastic Lineage

BMSC were grown in osteoinductive medium [62], that is, DMEM Low-glucose containing 10% FBS, 50 µM ascorbic acid, 10 mM β-glycerophosphate, 10^−8^ M dexamethasone (all from Sigma-Aldrich) and antibiotics. Cells were maintained in osteoinductive medium for one week with alternate day media exchange.

### Development of Simple and Mixed Spheroid Cell Culture Systems

Spheroids were developed as previously reported [53]. Briefly, 200 µL of 1% agarose (Invitrogen Corporation, Carlsbad, CA) were added per well of a 96 well round bottom plates and immediately removed. The plates were allowed to dry at RT, and 6.25–50×10^3^ BMSC were distributed per well. After 4 and 11 days in culture, spheroids were examined under an inverted microscope equipped with a digital camera. Their diameter in X and Y axis was measured with the help of a millimeter scale ruler. To avoid differences in spheroid diameter, simple spheroids were done with 2.5×10^4^ non-induced BMSC (simple non-induced spheroids) or one week-osteo-induced BMSC (simple osteo-induced spheroids) per each well while mixed spheroids were developed as following. Initially, 1.25×10^4^ of osteo-induced BMSC were plated per each well, and after 3–4 days, the same amount of non-induced BMSC was plated onto pre-formed osteo-induced spheroids.

### Cell Tracing

Osteo-induced BMSC were stained with the fluorescent tracker CM-DiI (indocarbocyanines) and CD34^+^ cells were labeled with CFSE (5-(and 6-)-carboxifluorescein diacetate succinyl ester) both from Invitrogen. Staining was done according to manufacture's instructions.

### Coculture of CD34^+^ Cells with BMSC Spheroids

After the development of simple and mixed spheroids, 2–3×10^4^ CB or BM magnetically selected CD34^+^ cells were plated onto each spheroid and co-cultured in Iscove's Dulbecco's modified medium with 10% FBS for up to 7days. Cells in the supernatant were collected at intervals. Spheroids were vigorously washed three times to remove non-adherent cells and cells attached to their surface, and enzymatically dissociated with 0.125% trypsin (0.78 mM EDTA) associated with mechanic dissociation (pipetting). The content of CD34^+^ cells in the supernatant and spheroids was evaluated by flow cytometry and the proportion of cells that migrated into the spheroids was calculated as percentage of the total. To distinguish cell migration from proliferation, the supernatant was removed after 24 hours of co-culture and the spheroids were vigorously washed, maintained for up to 48 hours, and analyzed as described. For each time-point, pools of three spheroids in duplicate were analyzed. Cell proliferation was investigated by BrdU incorporation assay. Immediately after plating CB CD34^+^ cells onto spheroids, 100 ng/mL BrdU (Sigma-Aldrich) were added in the culture medium, and the medium was renewed every two days.

### Cell Sorting and Methylcellulose Culture

Colony assays were done after 2 and 7 days of co-culture with simple or mixed spheroids. Briefly, CD34^+^ cells from the supernatants and from enzymatically digested spheroids were sorted using a MoFlo™ (Dako Inc., Fort Collins, Co, USA). The sorted cells (300 cells / 25 mm dish) were then cultured in methylcellulose using MethoCult GF H4535 medium (Stem Cell Technologies, Vancouver, BC, Canada), and 4 U/mL rhEPO (Eritromax®, Blaüsiegel) were added. All cultures were maintained at 37°C in a humidified incubator with 5% CO_2_ in air. Differential colony counts were scored after 10 to 12 days by morphologic characteristics using an inverted microscope and confirmed by staining individual colonies with May-Grünwald-Giemsa (MGG).

### Ultrastructural Analysis

For Transmission and Scanning Electronic Microscopy (TEM and SEM) spheroids were fixed in 2.5% glutaraldehyde (Ted Pella Inc., Redding, CA) in 0.1 M Na-cacodylate (EMS, Electron Microscopy Sciences, Hatfield, PA) buffer (pH 7.2) for 1 hour on ice. After post-fixation for 30 min with 1% OsO_4_ (EMS), 3.8% potassium ferricyanide (Vetec Química Fina, Rio de Janeiro, RJ, Brazil) and 2.5 mM CaCl_2_ (Sigma Aldrich) in the same buffer, samples were dehydrated in a series of acetone (30%–100%), and finally embedded in Epon 812 (EMS) for TEM. Ultrathin sections were cut (Ultracut, Leica SUCT), collected in copper grids, stained with uranyl acetate and lead citrate (Vetec), and examined under a Zeiss EM 10C transmission electron microscope. For SEM, spheroids were dried at the critical point with CO_2_ (Critical Point Dryer, CPD 030, Balzers Union), mounted on aluminum stubs, and coated with 20 nm-thick layer of gold (MED 010, Balzers Union). The samples were examined with a Zeiss 940 DSM scanning electron microscope.

### Morphological Analysis

Spheroids were fixed in 4% paraformaldehyde (Vetec) and processed routinely for paraffin inclusion. Paraffin slices were rehydrated and stained with Hematoxilin-Eosin (HE), Von Kossa, and picrosirius modified for confocal microscopy [63]. Since eosin is a fluorescent green dye [64], H-E stained slices were also prepared for confocal microscopy. Immunohistochemistry was performed using Novolink™ Polymer Detection System (Novocastra Laboratories, New Castle, UK). After block of endogenous peroxidase, and recovery of antigen epitope with heated water bath, sections were incubated with the primary antibody overnight at 4°C. Sections were then washed and incubated with the Novolink™, according to manufacture's instructions. The reaction was developed using a solution of 3-3-diaminobenzidine (DAB) with hydrogen peroxide. Nuclei were counterstained with hematoxylin. For immunofluorescence, fixed spheroids were embedded in Tissue-Tek O.C.T. (Sakura Finetek USA inc., Torrance, CA) and snap frozen. Cryostat sections of 5-20 µm were treated with 0.01% (or 0.2% for cytoskeleton staining) Triton X (Sigma-Aldrich) for 15 minutes. Non-specific sites were blocked with PBS 5% bovine serum albumin (BSA, Sigma-Aldrich) for 1 hour. Sections were incubated for 16 hours at 4°C with primary unlabeled antibodies diluted in PBS 1% BSA, washed three times with PBS and incubated for 1 hour at RT with conjugated secondary antibodies. Finally, sections were incubated with 1 µg/mL of 4′,6′-diamidino-2-phenylindole (DAPI, Invitrogen-Molecular Probes). Slides were examined and images were captured using a LSM510META (Carl Zeiss, Jena, Germany).

### Detection of Apoptotic Cells

Apoptotic nuclei were detected by TdT-mediated dUTP terminal nick-end labeling kit (TUNEL, ApopTag® Plus Fluorescein in Situ Detection Kit, Chemicon Int., Inc., Temecula, CA). Nuclei were counterstained with 1 µg/mL DAPI. Slides were examined in a fluorescence microscope (Axiophot, Zeiss) and images were acquired with AxioCam Hrc digital camera. To determine the percentage of apoptotic cells, cell nuclei were counted within regions measuring 800 µm^2^ in the peripheral and central area of each spheroid slice.

### FACS Analysis and Cell Proliferation Assay

Cell suspensions were washed with PBS with 3% FBS and 0.1% sodium azide (staining buffer) and incubated for 30 min at 4°C with antibodies. Cells were washed with the staining buffer and carefully suspended in exactly 400 µL. Acquisition was performed on a FACSCalibur using the CellQuest 3.1 software (BD Bioscience), determining time as the parameter to limit acquisition [53]. Intracellular staining was performed after cell surface staining as previously described [65]. Analysis was done using the WinMDI 2.8 software (http://facs.scripps.edu.in/pub/pc).

### RT-PCR Analysis

Total RNA was extracted using TRIZOL reagent (Invitrogen) following the manufacturer's instructions. First-strand cDNA was prepared using oligo-dT primer (500 mg/mL, Invitrogen) and Moloney murine leukemia virus reverse transcriptase (M-MLV RT,200 U/mL, Invitrogen) following standard protocols. Briefly, to 2 µg of total RNA, 0.5 mg of oligo-dT was added, and the mixture was kept at 68°C for 4 min before the addition of 0.01 M of DTT (DTT 0.1 M, Invitrogen), nucleotides (dNTPs 25 mM, Invitrogen), and 200 U of M-MLV RT. The mix was sequentially incubated at 39°C and 90°C for 2 hour and 10 min, respectively, and stored at −20°C. The cDNA was used for PCR amplification with specific primers ANGPT1: 5′ACTGTGCAGATGTATATCAAGC3′, 3′GTGGAATCTGTCATACTGTGAA5′; CXCL12: 5′CCGCGCTCTGCCTCAGCGACGGGAAG3′, 3′CTTGTTTAAAGCTTTCTCCAGGTACT5′; DELTA1: 5′CCACGCAGATCACGAACACC3′, 3′TTGCTATGACGCACCATCC5′; DKK1: 5′TGGTCCAAGATCTGTAAACCTGTCC3′, 3′CTGGCTTGATGGTGATCTTTCTGTA5′; GAPDH: 5′ATCACCATCTTCCAGGAGCG3′, 3′CCTGCTTCACCACCTTCTTG5′; JAG1: 5′GACTCATCAGCCGTGTCTCA3′, 3′TGGGGAACACTCACACTCAA5′; KITLG: 5′GACAGCTTGACTGATCTTCTGGAC3′, 3′ACTGCTGTCATTCCTAAGGGAGCT5′; RUNX2: 5′CTCACTACCACACCTACCTG3′, 3′TCAATATGGTCGCCAAACAGATTC5′; SPP1: 5′CCCTTCCAAGTAAGTCCAACGAAAGC3′, 3′CTGGATGTCAGGTCTGCGAAACTTC5′ and WNT5a: 5′TTTTTCTCCTTCGCCCAGGTTGT3′, 3′GGCTCATGGCGTTCACCAC5′. The PCR was performed on a TGradient thermal gradient cycler (Biometra, Göttingen) in 30 µL containing 2 µL cDNA, 1.5 U Taq polymerase (Cenbiot Enzimas, Porto Alegre, RGS, Brazil), 2 mM MgCl_2_, 0.1 mM of dNTPs, and 0.4 pmol/mL of each primer. Samples were cycled for 1 min at 96°C, 45 seconds annealing at specific temperatures for each primer, and 1 min extension at 72°C with a final extension of 5 min at 72°C. Aliquots (5 µL) were collected after 34 cycles and loaded on a 1.2% agarose gel. Gels were stained with ethidium bromide (Sigma-Aldrich) and visualized under a UV transilluminator. Changes in gene expression were verified by semiquantitative analysis. Densitometry analysis of the agarose gel was normalized against GAPDH, as an internal standard. Negative control was done as described with a PCR-mix lacking cDNA.

### Statistical Analysis

Data were presented as mean ± standard error of the mean (± SEM). Gaussian distribution of samples was tested and the statistical significance of the data was evaluated using the two-tailed *t*-test. The P value is shown in figures and statistical significance was considered when p<0.05.
